# 

**Published:** 1880-01

**Authors:** 


### THE BISTOURY:

Thad S. Up de Graff, M. D., Editor & Proprietor.

### *CONTENTS FOR JANUARY, 1880.*

### CONTRIBUTIONS.

PAGE.

Take of the Leaven of Damnation......BEN. H. PRATT ........ 199

What We Seem and What We Are......AUSBURN TOWNER ........ 202

Specialties........J. J. KEYES ........ 205

Microscopic........S. O. GLEASON, M. D........ 207

Statistics........J. B. CHANDLER ........ 208

### MEDICAL ITEMS AND NEWS.

Typhoid Fever Propagated—Cough Mixture—Chloroform Cough Mixture—Pertussis—Sore Nipples—Goulard's Cerate—Acne Punctata—Anti-Spasmodic Mixture—Conical Cervix—Anti-Asmatic Powder—Hoarseness—Anti-Septic Balsam—Gout—Diphtheritic Poison—Carbuncle of the Urethra—Freckles—Hæmatoma of Douglass' Pouch—Gathered Breasts—Vaginitis—Psoriasis—Fissure of the Rectum—Treatment of Funis—Diarrhœa—Cough Mixture—Eruption from Chloral—Whooping Cough—Ice Cream and Beef Juice—Nervous Prostration—Reptile Poisons—Puerperal Fever—Fungous Vegetations of Endometrium—Cancer of the Cervix—Cardiac Palpitations—Habitual Constipation—Bronchitis—Ovariotomy—Perimetritis—Nitrate of Amyl—Prolapse of both Ovaries—Ovarian Cyst—Cystitis in Female—Diarrhœa—Experiments on the Living Brain—Infantile Colic—Baldness—Prize for Essay on Diphtheria—Whooping Cough Syrup—Insomnia—Whooping Cough—Diabetic Urine........209 to 218

### OUR CORRESPONDENCE.

Five Letters and Replies ........ 218

### HOUSEHOLD HINTS AND RECIPES.

Sealing Wax for Fruit Cans—To remove Ink from Carpets—Japanese Cement—To Plate Iron Castings with Copper—To protect Iron from Rust—Shoe Blacking—Cement for Metal and Glass—To Remove Spots from Varnish—Influence of Gas Light upon the Eyes—Soot Tea for Roses—Transparent Paper—Long-Made Beef Tea—To Clean Old Engravings........218 to 221

### EDITORIAL.

ADAM ........221

FAILING SIGHT ........222

CORONERS ........223

### CHIT-CHAT.

Subscriptions—Microscope—Adam Inscription—New Cover—Telegraph Boys—Penny Nuisance at School—That Salad Contest—A Free Love Marriage—Got the Best of Him ........224 to 227

